# Institutional Questions

**Published:** 1900-05-05

**Authors:** 


### INSTITUTIONAL QUESTIONS.

### Treatment of Walls.

"Chairman" writes: What do you recommend as the best wall surface finishing for the wards and operation room of a small country hospital. Expense is a very serious consideration, but the committee are anxious to do the best thing in the best way as far as possible at the first, and wish to ascertain what material is really most recommended from a sanitary point of view.

*** For your wards a good cement surface, painted and varnished, would probably yield as good results as any process that could be suggested. This admits of easy and thorough cleansing, and stands wear well. For the operating-room it would be well to have tiled walls, the ceiling being painted and varnished, so that every corner may be washable.

### Hospital Section at Earl's Court.

"Curious" asks: Can you give me any information about the Hospital Section, which I see is announced as one of the features of the coming exhibition at Earl's Court? I see the Special Appeal Committee of the Charing Cross Hospital is mentioned in connection with this section. Does this mean that the section is in the hands of the Special Appeal Committee altogether?

*** Yes; we understand that this section, which is likely to be a very interesting one, has been undertaken by the Charing Cross Hospital Special Appeal Committee. The secretary is Mr. R. G. Lund. Preparations for this section are not very far advanced as yet, but it is in good hands, and should give a very complete demonstration of hospital arrangements and appliances.

### Hospitals in British Columbia.

"Emigrant" asks: Will you kindly tell me what hospitals there are in British Columbia, and if you can give me particulars of any there may be in Vancouver, I shall be greatly obliged.

*** There are hospitals at the following places in British Columbia (of which you will find details in "Hospitals and Charities," Scientific Press): Atlin City, Barkerville, Esquimault, Kamloops, Nanaimo, New Westminster, Vancouver, and Victoria. The Vancouver City Hospital, which is under the management of the Board of Health, provides accommodation for 61 beds, terms of admission being 5 dols. a week, private patients, 12 dols. There is a resident medical officer.

